# A systematic review of distal medial collateral ligament Stener‐like lesion: Good clinical and functional outcomes of surgical treatment

**DOI:** 10.1002/ksa.12721

**Published:** 2025-06-29

**Authors:** Alberto Grassi, Riccardo D'Ambrosi, Giacomo Dal Fabbro, Federico M. Adravanti, Barbara Boer, Angelo V. Vasiliadis, Stefano Zaffagnini

**Affiliations:** ^1^ II Clinica Ortopedica e Traumatologica, IRCCS Istituto Ortopedico Rizzoli Bologna Italy; ^2^ Dipartimento di Scienze Biomediche e Neuromotorie (DIBINEM) University of Bologna Bologna Italy; ^3^ IRCCS Ospedale Galeazzi—Sant'Ambrogio Milan Italy; ^4^ Dipartimento di Scienze Biomediche per la Salute Università degli Studi di Milano Milan Italy; ^5^ Centre for Orthopaedic Surgery and Sports Medicine OCON Hengelo The Netherlands; ^6^ Department of Orthopaedic Surgery, Sports Trauma Unit St. Luke's Hospital Panorama Greece

**Keywords:** distal medial collateral ligament, MCL, surgical repair, reconstruction, Stener‐like lesion

## Abstract

**Purpose:**

This study aimed to systematically evaluate the existing literature to account for the clinical and functional outcomes, complications and rate of return to sports among patients treated for distal medial collateral ligament (MCL) lesions that are isolated or associated with other knee ligament injuries.

**Methods:**

A systematic review was conducted based on the Preferred Reporting Items for Systematic Reviews and Meta‐Analyses (PRISMA) guidelines. Included were studies that reported postoperative clinical and functional outcomes in patients who had undergone surgery for an isolated or combined distal MCL lesion for medial knee instability. The authors evaluated surgical technique, rehabilitation protocol, postoperative outcomes (Lysholm, IKDC and Tegner scores and valgus stress radiograph), and return to sports and complication rates across the included studies.

**Results:**

A total of 70 patients (55 male, 15 female) were included. The mean age at surgery was 26.9 ± 8.2 years. The mean time from injury to surgery was 3.9 ± 3.0 weeks, while the mean follow‐up was between 6 and 68 months, with a mean of 45.8 ± 21.4 months. A total of seven (10%) complications were reported and six (8.6%) revision surgeries. The mean Lysholm score on 27 (38.6%) patients was 87.2 ± 7.3, while IKDC2000 was reported only in one study was 62. Regarding return to sport, five articles reported a full return to sports activity after 6–9 months, while when return to sports was evaluated by Tegner reporting a mean value of 7.8 ± 2.4 (range: 3–10).

**Conclusion:**

Notwithstanding conflicting data, most patients who have undergone distal MCL lesion surgical treatment, either in isolation or associated with knee ligamentous injuries, achieve satisfactory clinical and functional outcomes, a low rate of postoperative complications, and a high rate of return to recreational sports.

**Level of Evidence:**

Level V, systematic review.

**Abbreviations**
ACL: anterior cruciate ligament
AMA: American Medical Association
IKDC: International Knee Documentation Committee
LARS: ligament augmentation and reconstruction system
LM: lateral meniscus
MCL: medial collateral ligament
MINORS: methodological index for non‐randomized studies score
MM: medial meniscus
MPFL: medial patella‐femoral ligament
PCL: posterior cruciate ligament
POL: posterior oblique ligament
PRISMA: preferred reporting items for systematic reviews and meta‐analyses
PRMM: posterior root medial meniscus
sMCL: superficial medial collateral ligament

## INTRODUCTION

**Figure 1:**
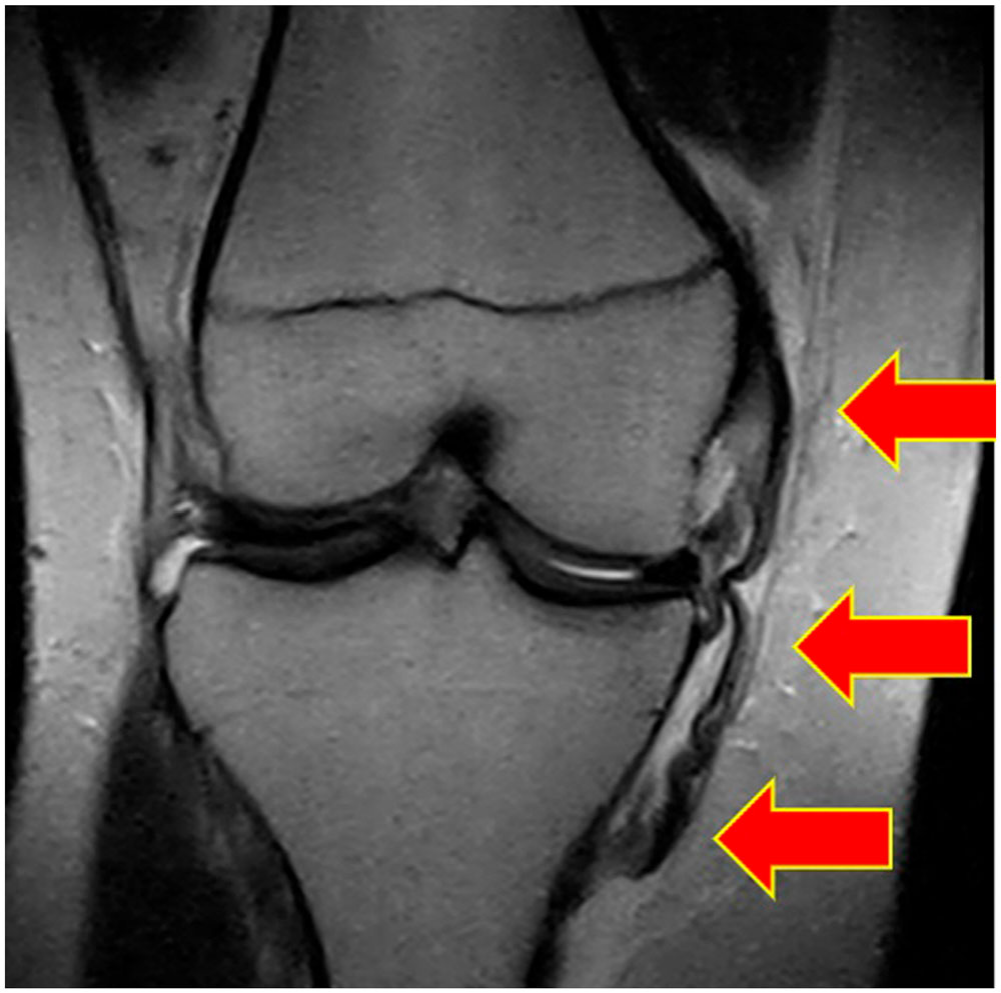
Magnetic resonance imaging, Coronal view. Red arrows show MCL in a right knee with a distal STENER‐like lesion. MCL, medial collateral ligament.

Medial collateral ligament (MCL) injuries are frequently observed in athletes and are one of the most prevalent types of ligament injuries in the knee [9]. Studies in the field of epidemiology focusing on professional football, rugby and National Football League athletes have revealed that injuries to the MCL make up approximately 26.9%–34.1% of all knee injuries in these sports. MCL injuries usually happen due to excessive strain on the ligament caused by a valgus force, either with or without a rotational load on the knee [20, 28]. Interestingly, superficial and deep MCL lesions are usually associated with injuries to other ligamentous structures in the knee such anterior cruciate ligament (ACL) [14, 17, 25]. These injuries most commonly affect the MCL femoral attachment or the ligament midsubstance. In a minority of cases, ranging from 5% to 19%, MCL injuries affect the distal tibial insertion [14, 23, 38, 39]. Injuries of distal MCL usually necessitate surgical intervention to restore the anatomy and facilitate the healing of the ligaments. However, due to the rarity of these injuries, research on the prognosis and outcomes of surgical treatment is restricted to case reports and small case series (Figure 1). Currently, there is a scarcity of evidence about the results of individuals who seek surgical treatment for grade III distal MCL injuries. These data would be highly important in assisting clinicians in making informed decisions for individuals who have this specific pattern of injury [1, 2, 6, 35].

This study aimed to systematically evaluate the existing literature to account for the clinical and functional complications and rate of return to sports among patients treated for distal MCL lesions that are isolated or associated with knee ligament injuries. We hypothesized that this procedure yields good results in terms of valgus laxity, functional patient‐reported outcomes and the rate of return to sports when used to treat medial knee laxity.

## MATERIALS AND METHODS

The current systematic review was performed following the Preferred Reporting Items for Systematic Reviews and Meta‐Analyses (PRISMA) guidelines [27].

### Eligibility criteria

The literature selected for this study was based on the criteria detailed below.

#### Study designs

Studies conducted using randomized controlled trials, controlled (nonrandomized) clinical trials, prospective and retrospective comparative cohort studies, case–control studies, case series and case reports were included in the current study.

#### Participants

Studies conducted on skeletally mature patients treated for distal MCL lesions and who were evaluated for a minimum follow‐up of 6 months were considered eligible for the current study.

#### Interventions

Studies that reported data on clinical, functional and radiological outcomes after the distal MCL treatment, isolated or with concomitant ligament surgery, to treat medial knee laxity were considered eligible for the current study.

#### Types of outcome measures

The outcome measures extracted from the studies were the knee laxity, Lysholm score, International Knee Documentation Committee (IKDC) subjective and objective scores, and Tegner activity level, rate of return to sports and rate of complications. The data from studies using stress radiographs to perform a quantitative assessment of the preoperative and postoperative medial stability were also extracted. Failure was defined as revisions or clinical failures based on the definitions provided in each study.

### Information sources and search

A systematic search for relevant literature was performed on the PubMed (MEDLINE), Scopus, Embase and Cochrane Library databases in December 2023 and updated in March 2025. The publication date was not considered an inclusion criterion. Two independent reviewers (R. D. and A. G.) assisted in conducting and validating the search. The search was performed using the following terms, combined with the Boolean operators ‘AND’ or ‘OR’: ‘medial collateral ligament’ OR ‘MCL’ AND ‘distal’ OR ‘tibial avulsion’ OR ‘distal avulsion’ OR ‘Stener type lesion’.

Only papers published in English were included.

### Data collection and analysis

#### Study selection

The retrieved articles were first screened by title and, if found relevant, then screened further by reading the abstract. After excluding studies not meeting the eligibility criteria, the entire content of the remaining articles was evaluated for eligibility. To minimize the risk of bias, the authors reviewed and discussed all the selected articles, references and articles excluded from the study. In case of any disagreement between the reviewers, the senior investigator (S. Z.) made the final decision. At the end of the process, further studies that might have been missed were manually searched for by going through the reference lists of the included studies and relevant systematic reviews.

#### Data collection process

The data were extracted from the selected articles by the first two authors using a computerized tool created with Microsoft Access (Version 2010; Microsoft). Every article was validated again by the first author before analysis. For each study, we extracted the data regarding the patients (age, sex, duration between injury and surgery and follow‐up evaluation), their injuries (type, origin and associated injuries), surgical technique (type of graft used, fixation technique and tensioning protocol), rehabilitation protocol, postoperative outcomes (Lysholm score, IKDC subjective and objective scores, knee laxity and Tegner), rate of complications, failures and rate of return to sports.

#### Level of evidence

The Oxford Levels of Evidence set by the Oxford Centre for Evidence‐Based Medicine were used to categorize the level of evidence.

#### Evaluation of the quality of studies

The quality of the selected studies was evaluated using the Methodological Index for Non‐Randomized Studies (MINORS) score. The checklist included 12 items, of which the last four were specific to comparative studies. Each item was given a score of 0–2 points. The ideal score was set at 16 points for noncomparative studies and 24 for comparative studies. On the other hand, case reports were evaluated due to the paucity of the literature present on Stener‐like lesions [33].

## RESULTS

**Figure 2:**
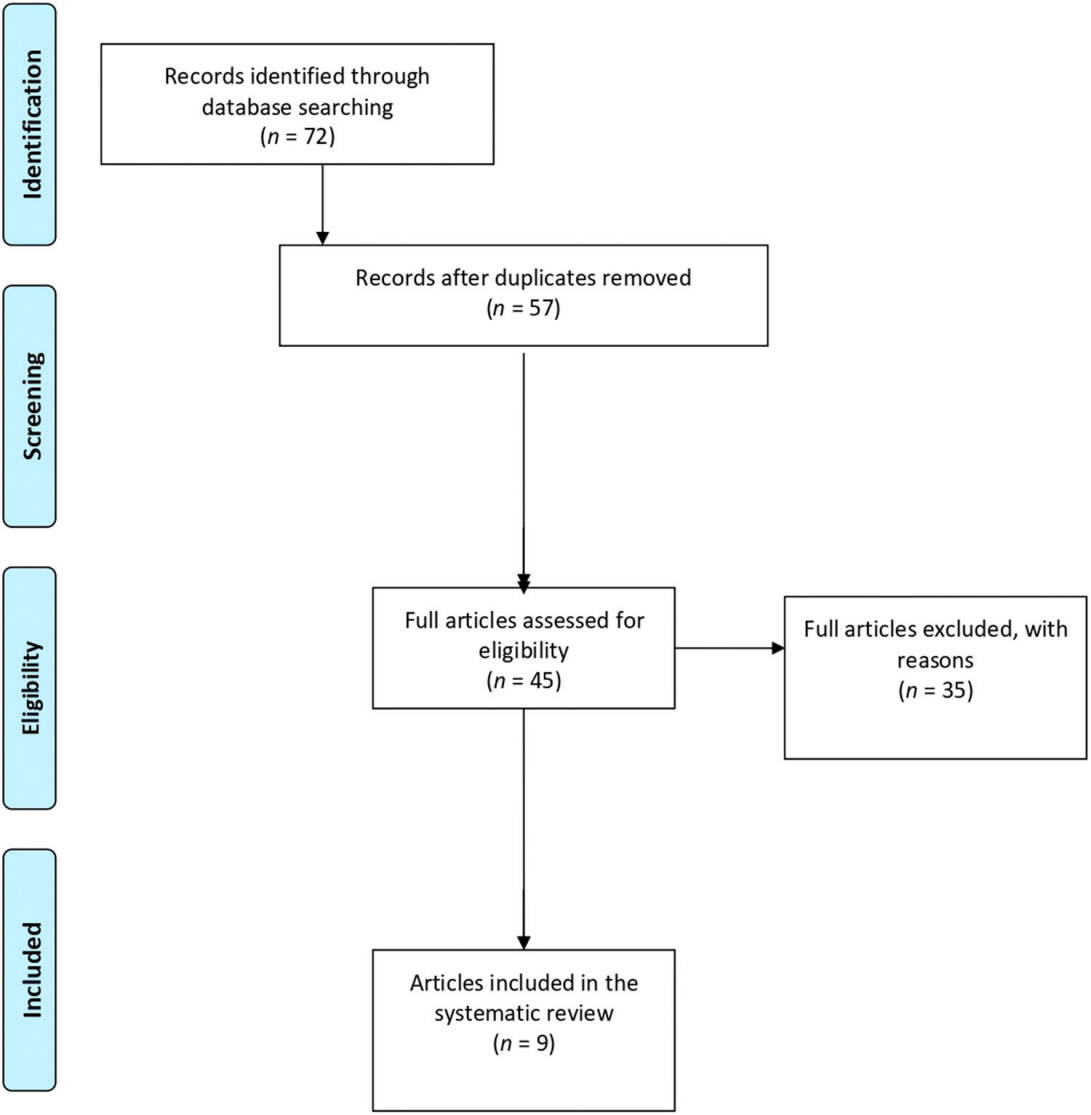
Flowchart depicting the selection process of articles included in the systematic review.

The electronic search yielded 72 studies. After removal of 15 duplicates, 57 studies remained, of which 12 were excluded after reviewing the abstracts, bringing the number down to 45. An additional 35 articles were excluded based on the aforementioned inclusion and exclusion criteria. No additional studies were found by manually searching the reference lists of the selected articles. This left nine studies for analysis [1, 7, 8, 10, 18, 21, 32, 35, 36]. Figure 2 shows the flowchart depicting the selection process for studies. The studies analyzed had a mean MINORS score of 12.4± 2.3 (excluding case report), which confirmed the methodological quality of the available literature (Table 1). Of the nine articles included in the review, four were grade V of the level of evidence and five were grade IV.

**Table 1:** Characteristics of the selected studies.

Lead author (year)	LOE	Minors score	Patients (M/F), *n*	Age, y, mean ± SD (range)	Time between injury and surgery, week	Follow‐up, mo mean ± SD (range)	Acute/chronic, n	Origin or mechanism of injury (*n*)	Associated injuries (*n*)	Grade of MCL injury
Desai et al. 2019 [10]	IV	13	20 (17/3)	22.7	5	68.2	20/0	n.a.	3 ACL + PCL 1 isolated MCL 16 ACL	3
Corten et al. 2010 [8]	V	n.a.	2 (1/1)	28.5	4	18	2/0	Skiing	1 ACL 1 isolated MCL	2
Kini et al. 2015 [21]	V	n.a.	1 (1/0)	20	3	12	1/0	Soccer	1 ACL	3
Shevate et al. 2021 [32]	V	n.a.	1(1/0)	25	3	24	1/0	Bumper Injury	1 PCL + PRMM	3
Thompson et al. 2023 [35]	IV	15	23 (20/3)	27.2	1.2	42	23/0	16 soccer 7 rugby	32 MCL isolated	3
Tiwari et al. 2018 [36]	V	n.a.	2 (1/1)	22	0.9	6	2/0	1 soccer 1 basketball	1 MCL isolated 1 ACL	3
Acevedo et al. 2021 [1]	IV	10	7 (7/0)	21	4	6	7/0	7 football	7 isolated	3
Jokela et al. 2021 [18]	IV	14	7(3/4)	49	11	66	7/0	5 high energy trauma 2 low energy trauma	3 meniscal lesion 5 chondral lesion	3
Brimmo et al. 2019 [7]	IV	10	7 (4/3)	23.85	n.a	48	n.a.	1 skiing 2 football 2 fell 1 trampoline 1 gymnast	2 ACL + MM 1 MPFL + POL + MM 1 ACL + LM + POL 1 ACL + LM + MM 1 ACL + LM 1 POL + MPFL + MM + partial PCL	6 grade 3 1 grade 2

Abbreviations: ACL, anterior cruciate ligament; LM, lateral meniscus; MCL, Abbreviation: LOE, level of evidence, medial collateral ligament; MM, medial meniscus; MPFL, medial patella‐femoral ligament; PRMM, posterior root medial meniscus; PCL, posterior cruciate ligament; POL, posterior oblique ligament.

### Patients' characteristics

A total of 70 patients were included of which 55 (78.6%) were male and 15 (21.4%) were female. The mean age at surgery was 26.9 ± 8.2 years. The mean time from injury to surgery was 3.9 ± 3.0 weeks, while follow‐up was between 6 and 68 months, with a mean of 45.8 ± 21.4 months. Except in one case, where the lesion was grade II, all other distal MCL lesions were grade III according to the Houston modified AMA classification. Concomitant lesion and mechanism of injuries are reported in Table 1.

### Surgical technique and rehabilitation protocol

The most common surgical treatment included open repair of the distal MCL (95.7% of cases), while a reconstruction procedure with allograft was performed in only three cases (4.3%). Different techniques were used to repair the distal MCL including anchors with different calibers and washer screws, highlighting no consensus for the surgical repair of ‘Stener‐like’ lesions. Moreover, ligament reinforcement with synthetic materials (e.g., Ligament Augmentation And Reconstruction System [LARS]) was performed in six cases (8.5%). Regarding the degree of knee flexion at ligament fixation, in three studies it was reported that the MCL was secured at 30° of flexion, while the other studies did not specify the degree of knee flexion for ligament fixation. Braces were used in most studies with knees fixed at either full extension or 30° of flexion. Three studies suggested not to apply weight on the operated knee and two suggested partial weightbearing. Surgical and rehabilitation protocols are reported in Table 2.

**Table 2:** Surgical technique of STENER‐line lesion repair and postoperative protocols of the included studies.

Lead author (year)	Surgical technique (in brief)	Tensioning protocol	Brace	Weight bearing	Passive and active exercise time
Desai et al. 2019 [10]	Surgical repair of the MCL involved ligamentous advancement followed by tibial fixation using a suture anchor and ligament washer technique. Dual suture anchors were placed distal to the joint line to recreate the deep meniscal tibial ligament. A bicortical screw with spiked ligament washer was used to create the distal insertion of the superficial MCL.	30° of flexion	n.a.	n.a.	n.a.
Corten et al. 2010 [8]	Two Mitek anchors (DePuy, Brussels, Belgium) were introduced into the bone and the nonabsorbable suture wires were sutured through the torn MCL with Bunnell‐type stitches	30° of flexion	4/5 weeks at 30°	No weight bearing for 3 weeks	Isometric strengthening from Day 1
Kini et al. 2015 [21]	Primary repair and reinforcement using fiber wire and distal tibial fixation with suture anchors	n.a.	For 6 weeks: 30° for a week 20° for a week 0° for 4 weeks	n.a.	n.a.
Shevate et al. 2021 [32]	The deep MCL was repaired primarily to its tibial attachment with 2‐0 Ethibond. The sMCL was advanced back to its tibial attachment site and repaired with a titanium 3.0 mm suture anchor. Using Stannard's modification of Kim's technique, sMCL was augmented with semitendinosus graft and fixed over the femoral attachment with 5.0 titanium suture anchor	n.a.	Straight knee brace for 4 weeks	No weight bearing for 4 weeks	Isometric strengthening from day 1
Thompson et al. 2023 [35]	Two suture anchors were placed proximally within the tibia just below the joint line, anteriorly and posteriorly, and one distally at the distal end of the MCL at its tibial insertional footprint. Each anchor had two nonabsorbable ultra–high molecular weight polyethylene fiber sutures, which were sutured into the free end of the avulsed ligament with a modified Kessler technique	30° of flexion	Brace locked in extension for 2 weeks	Partial weightbearing	n.a.
Tiwari et al. 2018 [36]	Repaired with internal bracing using Fibertape (Arthrex, Naples, FL) and suture anchors	n.a.	Knee brace for 3 and 6 weeks	n.a.	n.a.
Acevedo et al. 2021 [1]	Repair with double‐row suture technique	n.a.	Full extension for 2 weeks	Non‐weightbearing for 6 weeks	Isometric exercise
Jokela et al. 2021 [18]	Repair (*n* = 6) or reconstruction using modified Bosworth technique with tendon allografting (*n* = 1)	n.a.	Knee brace for 12 weeks	Partial weightbearing for 4 weeks	From day 1 isometric strengthening
Brimmo et al. 2019 [7]	Three open repair one open reconstruction with tibialis anterior graft three open repair with internal brace augmentation	n.a.	n.a.	n.a.	n.a.

Abbreviations: MCL, medial collateral ligament; sMCL, superficial medial collateral ligament.

### Clinical outcomes, return to sport, complications and revision surgery

A total of seven (10%) complications were reported of which two (2.9%) included flexion deficit >20°, three (4.3%) included persistent pain due to LARS material, one (1.4%) included neuralgia of saphenous nerve and one (1.4%) persistent instability.

A total of six (8.6%) revision surgeries were performed of which three (4.3%) for screws removal, one (1.4%) for LARS removal, one (1.4%) arthroscopic lavage and one (1.4%) MCL refixation.

Regarding postoperative knee ROM, only one study reported a mean postoperative knee ROM of 7°–135° while other four studies reported full ROM after surgery. Concomitantly laxity was analysed in 7 studies with 5 studies reporting no laxity post‐operatively, while one study analyzed valgus stress resulting in a 2.5 mm side‐to‐side difference after surgery.

Regarding clinical outcomes, mean Lysholm score on 27 (38.6%) patients resulted 87.2 ± 7.3, while IKDC2000 was reported only in one study was 62.

Regarding return to sport, five articles reported a full return to sports activity after 6–9 months, while when return to sports was evaluated by Tegner reporting a mean value of 7.8 ± 2.4 (range: 3–10).

All these data are reported in detail in Table 3.

**Table 3:** Data regarding clinical outcomes, return to sport, complications and revision surgeries.

Lead author (year)	ROM	Laxity	Clinical outcomes	Complications	Revision surgery	Return to sport
Desai et al. 2019 [10]	7°–135°	no laxity at 0‐ degree and < 5 mm laxity at 30° flexion)	LYSHOLM 91.5 IKDC 88.8	Grade I MCL sprain	None	TEGNER 7
Corten et al. 2010 [8]	Full	No Laxity	n.a.	None	None	Complete resume
Kini et al. 2015 [21]	Full	No Laxity	n.a.	None	None	Full at about 6–9 months
Shevate et al. 2021 [32]	Full	No Laxity	n.a.	None	None	Complete resume
Thompson et al. 2023 [35]	n.a.	n.a.	n.a.	1 saphenous nerve nevralgia 3 persistent pain due to LARS material	2 LARS screws removal 1 LARS removal	Tegner 10
Tiwari et al. 2018 [36]	Full	No laxity	n.a.	None	None	n.a.
Acevedo et al. 2021 [1]	n.a.	No laxity	n.a.	None	None	Complete resume
Jokela et al. 2021 [18]	n.a.	Valgus stress 2.5 mm	IKDC2000 62 Lysholm 75	2 flexion deficit (>20°)	1 arthroscopic lavage 1 MCL refixation	Tegner 3
Brimmo et al. 2019 [7]	n.a.	n.a.	n.a.	1 instability	1 screw remove due to pain	n.a.

Abbreviations: LARS, Ligament Augmentation and Reconstruction System; MCL, medial collateral ligament; ROM, range of motion.

## DISCUSSION

The most important finding from the current systematic review is that the literature available is scarce with only five case series with a limited number of patients (excluding case reports) reporting mid‐term clinical results and different surgical approaches (reconstruction vs. repair) and different grafts used.

Included studies indeed were too heterogeneously designed to serve this purpose, including small and different sample sizes, nonconsecutive groups of patients and using incomplete and different methods to quantify the clinical outcomes after surgical treatment of distal MCL. Despite the support of the scientific literature, there is a necessity to better understand the ideal treatment for distal MCL lesions.

**Figure 3:**
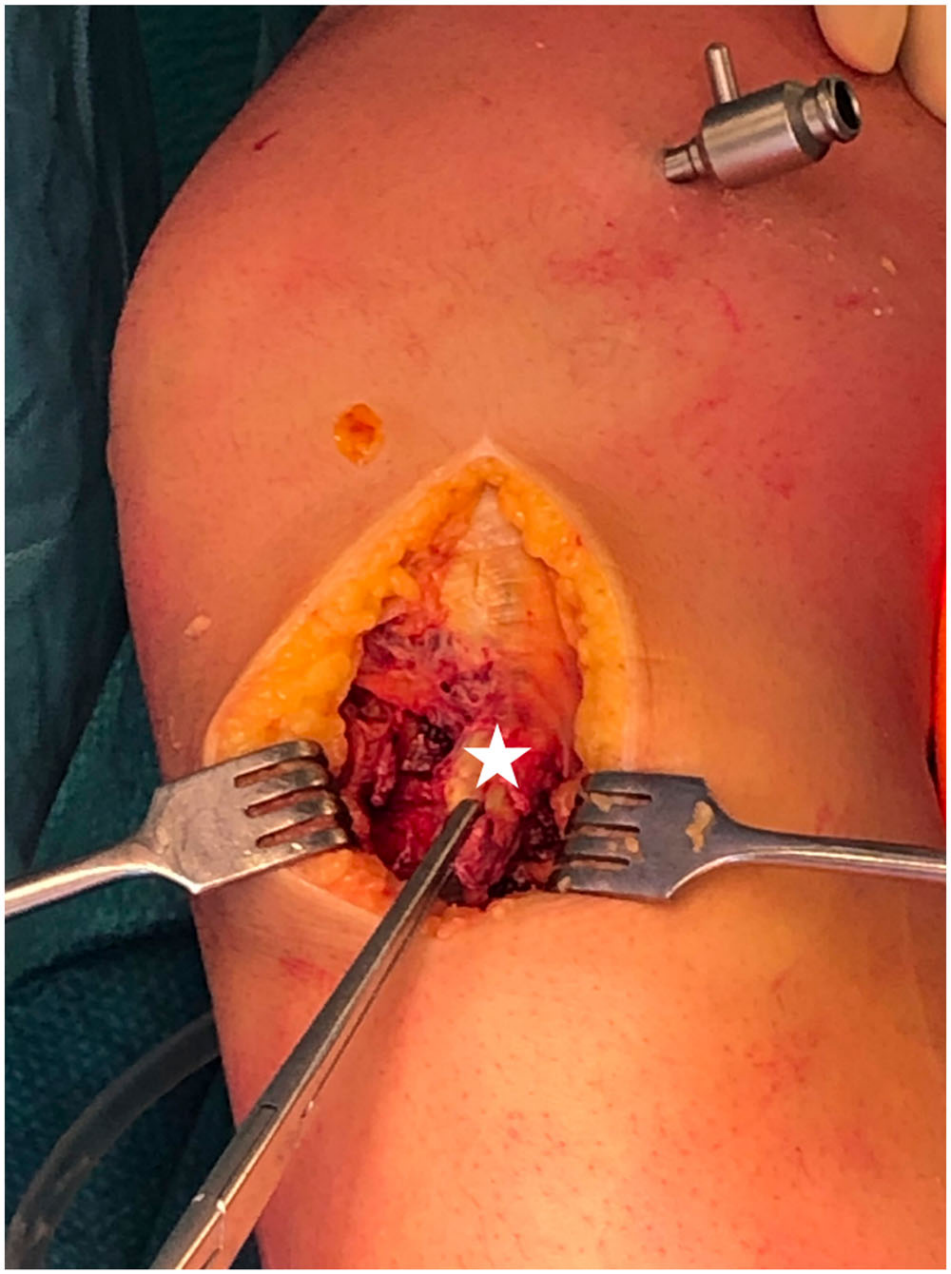
Right knee, the asterisk shows the intraoperative view of distal MCL avulsion being fixed with a suture anchor to the tibia. MCL, medial collateral ligament.

Being MCL and antero‐medial retinaculum important structures to stabilize the knee during tibial rotation and anterior translation [15], the most common surgical technique included open repair with suture anchors. However, the exact placement in order to recreate the anatomy of the MCL with the correct reinsertion of its distal superficial fibers was not reported in detail and was inconsistent among the included studies (Figure 3).

Therefore, no recommendations can be made regarding a gold‐standard technique for the management of these lesions. The augmentation with synthetic material is a matter of debate as well [19] in the setting of ligament repairs: however, the reinforcement with synthetic devices has been used only in a minority of the included studies. Thus, it could not be recommended as a mandatory procedure in the treatment of distal MCL ruptures.

Notwithstanding conflicting data, most patients who have undergone distal MCL lesion surgical treatment, either in isolation or associated with knee ligamentous injuries, achieve satisfactory clinical and functional outcomes, a low rate of postoperative complications and a high rate of return to recreational sports.

Recognizing a distal MCL lesion is crucial when dealing with severe MCL injuries in order to properly guide treatment. Nonoperative treatment is commonly used for grade I or grade II MCL (according to Hughston Modification of AMA classification) injuries that occur along with acute ACL ruptures. After the MCL has healed and knee stability has been restored, ACL reconstruction is generally performed. There is a debate about the optimal treatment for acute grade III MCL injuries that happen at the same time as ACL ruptures [12, 26, 34]. Six studies in the literature, involving a combined sample size of 185 individuals, recommended the surgical reconstruction of the ACL and nonoperative therapy of the MCL [3, 16, 24, 29, 30, 31]. Grant et al. conducted a review suggesting that ACL restoration should be carried out within a subacute timeframe, once full motion has been restored. Valgus instability should be evaluated at that point and patients with persistent valgus instability should have MCL repair or reconstruction. Nevertheless, these studies failed to take into account the presence of rotatory instability resulting from deficiencies in the ACL and MCL [13]. In accordance with Grant et al., Dong et al. support that ACL and MCL surgeries should be conducted within a subacute timeframe [11, 13]. This is because acute anatomical repair has proven to be ineffective in achieving sufficient rotatory stability and has resulted in a higher occurrence of medial pain, as demonstrated in our study [11]. Furthermore, the clinical outcomes of triangular ligament reconstruction in this study did not exhibit a substantial disparity when compared with our prior work including the repair of the MCL in patients with chronic injuries [11]. The results of the present study revealed a low rate of complications and failure rate, regardless the surgical technique used for distal MCL lesions; surgeons employed diverse fixation methods in the aforementioned approaches, selecting them according to their personal choice and expertise. The majority of authors utilized a suture anchor. Nevertheless, satisfactory outcomes have been achieved using alternative methods or graft such as LARS, staples or augmentation with hamstring tendons. Overall, the use of allograft, as reported by Dong et al. carries the possibility of problems such as infection and biomechanical degradation caused by irradiation. It also incurs extra surgical expenses and may not be accessible in certain countries [11]. The process of harvesting autografts, such as hamstrings, can increase the severity of damage to nearby tissues, increase the risk of complications at the donor site and potentially weaken the dynamic medial stabilizers when utilizing knee flexor tendons [22].

In the current systematic review, the analysis was performed on the clinical and functional outcomes of distal MCL lesions, and the results are in line with updated literature as animal studies demonstrated the remarkable healing ability of solitary MCL injuries when protected range of motion, protected weightbearing and regulated training are implemented early in the care. Studies conducted on rabbits have demonstrated that early restoration of the ACL in the knee can potentially avoid late valgus instability and promote superior healing of MCL injuries. In contrast, primary repair of the MCL following ACL reconstruction has shown limited or no improvement in MCL healing. The decrease in in situ stresses in the MCL following ACL restoration may help to clarify the enhanced healing of the MCL observed in laboratory investigations using this treatment protocol [5]. Biomechanical investigations conducted on deceased human bodies have demonstrated that when the MCL is injured, the ACL assumes the main role in preventing inward rotation of the knee joint [40]. Additionally, these two ligaments act as secondary restraints to support each other [4].

Analyzing return to sports, promising results were found with a high percentage of patients that returned to their sports activity, without any kind of limitations, in particular, it is interesting to analyze the population treated by Thompson et al. being all professional athletes. All participants were professional athletes with magnetic resonance imaging (MRI) confirmation of complete rupture with Stener‐like lesion, and thus, a nonoperative pathway was not attempted given the risk of failure to return to preinjury sporting level. The decision was made on first principles. With conservative treatment, there is a high risk of residual MCL laxity after, and this triggered a desire to restore anatomy and early stability [35].

Finally, various timings for surgical treatment have been reported, with still no established consensus in the current literature on the optimal approach; early surgical intervention effectively reduced the development of scar tissue or adhesions, which could lead to tethering with nearby structures. Nevertheless, Thompson et al. reported three cases in which it was necessary to extract LARS screws or synthetic tape due to the presence of pain and noticeable synthetic material [35]. Frequent instances of medial tibial screw removal are often recorded following ACL repair due to localized pain. The additional synthetic material needed for LARS augmentation was inserted in a superficial subfascial area of the knee. This may have caused more inflammation and the creation of scar tissue after the injury, which was observed after the removal process [37].

There are multiple limitations in this study. The literature research uncovered significant heterogeneity among the studies examined in terms of the range of injuries treated, the time elapsed between injury and surgery, and the variations in procedures used (such as the type of graft and fixation methods). Specifically, the participants in each research had medial knee laxity resulting from various injuries, including isolated grade 3 MCL injuries, ACL and/or posterior cruciate ligament (PCL) tears, and unsuccessful prior surgeries for multiligament injuries. The presence of heterogeneity in the data may complicate the interpretation of the results.

Another limitation is the methodological rigor of the chosen studies. The majority of the research consisted of retrospective case series with a diverse variety of individuals, but lacked a control group. This significant methodological constraint emphasizes the necessity for additional meticulously planned prospective research and deeper exploration of the topic.

The clinical significance of this systematic review is that a combined reconstruction of the cruciate ligament and distal MCL lesion is increasingly acknowledged as an effective treatment for high‐energy multiligamentous injuries associated with grade 3 MCL injuries. This is true both in general and specifically among athletes. According to the latest evaluation, this method seems to be both safe and consistent, yielding satisfactory clinical and functional results during the follow‐up period. Nevertheless, additional rigorous investigations are required to verify the accuracy of the current results.

## CONCLUSION

There is a paucity of literature regarding the surgical treatment and outcomes of distal MCL tears. The few included studies reported different surgical techniques of repair or reconstruction with or without synthetic augmentations, included small and different sample sizes, nonconsecutive groups of patients and used incomplete and different methods to quantify the clinical outcomes after surgical treatment of distal MCL. Notwithstanding conflicting data, most patients who have undergone distal MCL lesion surgical treatment, either in isolation or associated with knee ligamentous injuries, achieve satisfactory clinical and functional outcomes, a low rate of postoperative complications, and a high rate of return to recreational sports.

## AUTHOR CONTRIBUTIONS

Alberto Grassi and Stefano Zaffagnini had the initial idea of the study and supervised the work by reviewing the paper and conducting the decision‐making processes. Angelo V. Vasiliadis, Barbara Boer, and Riccardo D'Ambrosi contributed to the writing of the material and methods, results and discussion and the statistical analysis. Federico Maria Adravanti and Giacomo Dal Fabbro contributed to the article by making the articles selection and writing the introduction and abstract.

## CONFLICT OF INTEREST STATEMENT

Stefano Zaffagnini is a consultant for De Puy Synthes and Smith&Nephew and Editor in Chief of JEO journal. Angelo V. Vasiliadis is a member of the editorial board of JEO journal. The remaining authors declare no conflicts of interest.

## ETHICS STATEMENT

The authors have nothing to report.

## Data Availability

All data used for this study were publicly available on databases: PubMed (MEDLINE), Scopus, Embase and Cochrane Library.
